# The pregnancy outcome in patients with minor β-thalassemia

**Published:** 2011

**Authors:** Sedigheh Amooee, Alamtaj Samsami, Jamileh Jahanbakhsh, Mehran Karimi

**Affiliations:** 1 Department of Obstetrics and Gynecology, Shiraz University of Medical Sciences, Shiraz, Iran.; 2 Hematology Research Center, Nemazee Hospital, Shiraz University of Medical Sciences, Shiraz, Iran.

**Keywords:** *Minor β-thalassemia*, *Pregnancy outcome*, *Gestational diabetes mellitus*, *Oligohydramnios*

## Abstract

**Background: **β-thalassemia is the most common hereditary disease in Iran and more than 2 million carriers of the β-thalassemia mutant gene are living in this country.

**Objective: **To determine pregnancy outcome of women with β-thalassemia minor.

**Materials and Methods:** In this retrospective, case-control study in two universities affiliated hospitals in Shiraz, all pregnancies occurred between 2006 and 2008 were included. Patients were divided in two groups regarding the presence of β-thalassemia minor. Patients in case and control groups were matched according to maternal age, gestational age and number of previous pregnancies. Cesarean delivery, hypertensive disorders, gestational diabetes mellitus, premature rupture of membranes and preterm labor were recorded in each group and were compared using the χ^2^ or Fisher exact tests.

**Results:** Overall 510 β-thalassemia minor subjects and 512 healthy controls were studied. Cases with β-thalassemia minor had significantly higher prevalence of oligohydramnios (p<0.001) and cesarean section delivery (p=0.001). There was no significant difference regarding Apgar score in 1^st^ (p=0.65) and 5^th^ minute (p=0.25), IUGR (p=0.073), gestational diabetes mellitus (DM) (p=0.443) and preeclampsia (p=0.116) between two study groups.

**Conclusion: **β-thalassemia minor does not significantly influence the pregnancy outcome in the negative way.

## Introduction

The prevalence and severity of the thalassemia syndromes are population dependent (1). Thalassemia minor results in a variable degree of the disease but, depending on the rate of β-chain production, usually presents as asymptomatic anemia of mild degree (2). β-thalassemia is the most common hereditary disease in Iran and more than 2 million carriers of the β-thalassemia mutant gene are living in this country. 

About 110 mutant genes have been recognized all over the world of which 21 have been identified in the Iranian population. The mutant genes and their frequencies vary greatly in different parts of Iran. Prevalence of β thalassemia minor among Iranian is about 7% (3).

β-thalassemia minor represents the heterozygous state. In general, a heterozygote for thalassemia is diagnosed with a mild anemia (hemoglobin A level 1 or 2 g below normal range), low mean cell volume, low mean corpuscular hemoglobin, elevated hemoglobin A2, and normal or elevated hemoglobin F. During pregnancy, women with thalassemia minor will often show more significant anemia, which is often most prominent during the latter half of the second trimester and early third trimester (4-7). Thalassemia syndromes constitute a group of inherited hemoglobinopathies that require close maternal and fetal surveillance during pregnancy, including appropriate consultation with maternal fetal medicine and hematology specialists. Even for the women who are asymptomatic before pregnancy, the added stresses of pregnancy on the hematopoietic system can cause deterioration of maternal status. Health care providers must appreciate that the more severe the thalassemia syndrome, the more significant the consequences for both woman and fetus (4). 

The impaired globin synthesis impairs oxygen transport and delivery to tissues, placental bed, and fetus because of limited hemoglobin-oxygen binding. Furthermore, in more severe disease states, the accumulation of iron stores in vital maternal organ systems can lead to chronic organ dysfunction, such as cardiomyopathy and diabetes. These women must be monitored closely for worsening anemia and the development of pregnancy-associated complications (8).

Because of high prevalence of β-thalassemia in Iran and its possible effects on pregnancy outcome and limited study about this problem, we performed this retrospective case-control study to investigate pregnancy outcome of patients with β-thalassemia minor referring to Hafez and Zeinabieh Hospitals of Shiraz University of Medical Sciences between 2006 and 2009. These results can alert health care providers to take more care about β-thalassemia and pregnancy.

## Materials and methods

This was a case-control study which included all the pregnancies (7290) referring to Hafez and Zeinabieh Hospitals of Shiraz University of Medical Sciences between 2006 and 2008. The patients were divided into two groups regarding the presence of β-thalassemia minor. Patients in case and control groups were matched according to maternal age, gestational age and number of previous pregnancies. The study was approved by Shiraz University of Medical Sciences ethical committee and all the patients gave their written consents. The data was collected by a means of a questionnaire. Overall 510 β-thalassemia minor subjects and 512 healthy controls were enrolled in the study. Only women with singleton pregnancy were included. Patient with recurrent abortions (2 or more consecutive pregnancies resulting in spontaneous abortion), history of neural tube defect in previous pregnancies, history of infertility, history of any medical problem, undelivered in this center or mean follow-up visits less than three times were excluded. Follow-up intervals in case and control groups were similar.

Gestational age was calculated from the first day of last menstrual period or according to the sonography which was performed in the first months of pregnancy. Anemia was determined on the basis of hemoglobin less than 10 mg/dl. Anemia evaluation was performed in all the cases. Complete blood count (CBC), Iron studies (iron, total iron binding capacity) were performed for excluding iron deficiency anemia and the anemia of chronic disorders. Thalassemia was diagnosed according to CBC and standard hemoglobin electrophoresis criteria in the first trimester: an elevation of Hb A2 (equal or more than 3.5%) demonstrated by electrophoresis and column chromatography confirms the diagnosis of β-thalassemia trait. Blood transfusions were given only when hemoglobin (Hb) dropped significantly (less than 7mg/dl) either due to pregnancy or any other causes. Hemoglobin increased at least to 10 mg/dl with transfusion. According to American College of Obstetricians and Gynecologists criteria (9), folate supplement in each group (case and control) was given 1mg orally daily. 

In the study period each patient was evaluated for maternal age, parity, gestational age, maternal anemia during pregnancy (hemoglobin less than 10 g/dL) and birth weight. Poor pregnancy outcomes or complications included hypertensive disorders, gestational diabetes mellitus, premature rupture of membranes (rupture of membrane under 37 weeks gestational age) and preterm labor (labor pain under 37 weeks of gestational age). Hydramnios (amniotic fluid index more than 24 cm), oligohydramnios (amniotic fluid index 5 cm or less) and intrauterine growth restriction (IUGR) (when intrauterine growth under 10% by serial sonography especially in third trimester of pregnancy) were also evaluated using sonography in the third trimester. Labor and perinatal outcome included; placental abruption, meconium-stained amniotic fluid, cesarean delivery, Apgar score at 1 and 5 minutes less than 7, perinatal mortality, postpartum hemorrhage, maternal packed-cell transfusions, and neonatal ICU admission. 

**Statistical analysis**

Statistical analysis was performed with the SPSS package. Statistical significance was calculated by using the χ^2^ or Fisher exact test. We wanted to determine independent risk factors in pregnant women with beta-thalassemia minor, so a multivariate logistic regression model, with backward elimination, was constructed to find independent risk factors associated with maternal β-thalassemia minor. Odds ratios (ORs) and their 95% confidence intervals (CIs) were computed. A value of p-value less than 0.05 was considered statistically significant. 

## Results

We recorded 510 alive-births and 7 (1.4 %) stillbirths in case and 512 alive-births and 5 (1%) stillbirths in control group. The Hb A2 level in our cases usually was approximately 4-6%. Baseline characteristics of subjects in two study groups are shown in Table I. There was no statistically significant difference between these groups regarding these baseline characteristics. 

There was no significant difference between these groups regarding pregnancy hypertension and gestational diabetes mellitus (DM) (Table II). Polyhydramnios [Amniotic fluid index (AFI)>24cm] was observed in 1.4% of cases and 1.9% of control group while oligohydramnios was observed in 10.8% of case and 5.4% of control group (p=0.001, Table II). 

Regarding the perinatal complications, 3.1% of case group and 1.5% of control group developed IUGR (p=0.073). Cesarean delivery was significantly more common in case group than control group (38.3% vs. 26.5%; p=0.001, Table III). There was no significant difference between two groups regarding the Apgar score in 1^st^ (p=0.65) and 5^th^ minute (p=0.25), ICU admissions (p=0.17) and placental abruption (p=0.42). 

Using a multiple logistic regression model of risk factors for minor β-thalassemia, oligohydramnios, placental abruption and meconium-stained amniotic fluid were significantly associated with cesarean delivery (Table IV). 

**Table I:** Baseline characteristics of the two study groups

**p-value**	**Control group (n = 512)** **% (no** * **)**	**Case group (n = 510)** **% (no** * **)**	
			Maternal age
0.324	6.6% (34)	5.80% (30)	<19 years
0.322	90.3% (462)	89.4% (456)	19-35 years
0.204	3.1% (16)	4.8% (24)	≥ 36 years
			Gestational age
0.010	8.7% (45)	10.4% (53)	<36 weeks
0.010	65.8% (337)	69.2% (353)	36-39 weeks
0.006	25.5% (130)	20.3% (104)	≥ 40 week
			Parity
0.094	63.4% (324)	59.6% (304)	1
0.083	36.2% (186)	38.9% (168)	2-4
0.112	0.4% (2)	1.5% (8)	≥ 5
			Birth weight
0.180	11.6% (60)	15.5% (79)	<2500 grams
0.179	87.0% (445)	83.0% (423)	2500-4000 grams
0.108	1.4% (7)	1.5% (8)	>4000 grams
			Maternal anemia
<0.001	2.1% (11)	45.223% (230)	Hemoglobin < 10 mg/dl

* Number of patients

**Table II:** Obstetric risk factors of subjects with and without β-thalassemia minor

**p-value**	**Control group (n = 512)** **% (no** * **)**	**Case group (n = 510)** **% (no** * **)**	
0.116	6.8% (35)	4.8% (24)	Preeclampsia
0.434	3.9% (20)	3.5% (16)	Gestational diabetes
0.313	1.9% (10)	1.4% (7)	Hydramnios
0.001	5.4% (28)	10.8% (55)	Oligohydramnios
0.073	1.5% (8)	3.1% (16)	Intrauterine growth restriction
0.322	4.6% (23)	3.9% (20)	Premature rupture of membranes
0.100	4.1% (21)	6.00% (30)	Preterm labor

* Number of patients

**Table III:** Labor complications and outcomes of subjects with and without ß-thalassemia minor

**p-value**	**Control group (n = 512)** **% (no)**	**Case group (n = 510)** **% (no)**	
0.42	2.7% (14)	2.3% (12)	Placental abruption
0.500	6.00% (31)	5.80% (30)	Meconium-stained amniotic fluid
<0.001	26.5% (136)	38.3% (198)	Cesarean delivery
0.38	1.0% (5)	1.4% (7)	Stillbirth rate
0.002	1.4% (7)	4.4% (22)	Packed-cells transfusion
0.17	5.0% (26)	6.6% (34)	ICU admission
0.65	4.2% (22)	3.7% (19)	Apgar score at 1 min<7
0.25	0.7% (4)	1.4% (7)	Apgar score at 5 min<7

* Number of patients

**Table IV:** Multiple logistic regression model of risk factors for cesarean delivery

	**Odds ratio**	**95% Confidence interval**	**p-value**
Intrauterine growth restriction	0.85	0.33 - 2.2	0.74
Oligohydramnios	3.3	1.9 - 5.6	<0.001
Abruption	3.7	1.7 - 8.6	0.002
Thalassemia minor	1.6	1.3 - 2.2	<0.001
Meconium-stained amniotic fluid	5.4	3.06 - 9.5	<0.001

## Discussion

 Thalassemia is common in Iranian population. This case-control study was performed on patients with β-thalassemia minor to determine the maternal and fetal outcomes and describe most risk factors associated with β-thalassemia minor during pregnancy. This study consisting 517 β-thalassemia minor subjects is one of the biggest studies of its kind in Iran. Perinatal mortality (p=0.38) and Apgar score at 1min (p=0.65) and 5 min (p=0.25) after delivery were similar in patients with and without minor β-thalassemia minor. This is consistent with previous studies (9-13).

Thalassemia has been associated with an increased incidence of obstetrical complications (14). Adverse pregnancies outcomes detected in these case series, especially low birth weight (Wt < 

2500gr, 15.5% in case vs*.* 11.6% in control group), IUGR (3.1% in case vs. 1.5% in controls) and preterm delivery (10.4% in case vs. 8.7% in controls), but these finding were not statistically significant. Chronic maternal anemia during gestation might lead to fetal hypoxia, and predisposing the fetus to IUGR (1, 14). Thus, it was suggested that hemoglobin concentration should be maintained above 10 g/dL during these pregnancies (15). No significant association was found between hemoglobin levels and IUGR among thalassemic women in Eyal Sheiner and colleagues’ study and suggested that a different mechanism is responsible for IUGR in thalassemia minor patients (16). At least one study showed acute splenic infarct in β thalassemia minor (17). This mechanism may cause placental infarction but this theory needs further study to be approved.

In another study all adhesion molecules and CRP (C-reactive protein) increased in patients with thalassemia intermediate (18). Therefore this molecules and inflammation may cause placental insufficiency in β thalassemia minor but more study is needed.

Sheiner and associates (2004) reported that oligohydramnios were increased twofold in 261 affected women. In our study oligohydramnios was found in 10.8% of case and 5.4% of control group (p<0.001). Oligohydramnios is associated with IUGR and might be part of the relative hypoxemic state (16).

We found no statistically significant difference in the pregnancy outcome, preterm delivery, birth weight, growth restriction, pregnancy induced hypertension and gestational diabetes between thalassemic and non thalassemic patients. All studies investigating pregnancy outcome of patients with β-thalassemia minor found higher rates of cesarean delivery (11, 14, 15, 19). Likewise, in our study we found significantly higher rates of cesarean delivery.

By using a multiple logistic regression model of risk factors for minor thalassemia, in these pregnancies complications were significantly associated with cesarean delivery. However, IUGR was not statistically independent risk factor for cesarean delivery, unlike previous study that cesarean birth due to fetal distress related to fetal growth restriction (13).

In conclusion, hemoglobinopathies can be associated with a variety of effects on the mother, fetus or newborn. The effects range from absence of clinical disease to severe morbidity and death. Through the obstetrician-gynecologist's high index of suspicion based on clinical history and a close working relationship with a consultant hematologist, pregnancy outcome in patients with these disorders can be improved. 

Since most hemoglobinopathies are inherited as autosomal recessive conditions, screening, counseling, and prenatal diagnosis are important components of prenatal care for these women. Thalassemia syndrome, including β-thalassemia minor during pregnancy can present unique management challenges and requires close maternal and fetal surveillance. The pregnancy outcome in patients with beta-thalassemia minor, like prenatal outcomes, is not different from normal group. In spite of an attempt to keep hemoglobin levels above 7.0 g/dl, the incidence of fetal growth restriction and preterm birth has been relatively high, though maternal complications are rather not different from general. Care for such pregnancies should be multidisciplinary, incorporating a maternal–fetal medicine specialist, a genetic counselor, and a hematologist. However, since fetal growth restriction complicates more pregnancies with thalassemia syndrome, the need for close antenatal follow-up and frequent sonographic assessment of fetal growth can be overemphasized. 

Further prospective studies among high-risk populations for β-thalassemia with larger sampling should investigate the efficacy of such surveillance programs. The American College of Obstetricians and Gynecologists recommends screening for β-thalassemia in couples of Mediterranean ancestry.

We might miss some pregnant ladies with minor thalassemia and normal hemoglobin who were included in control group.

## Conclusion

 β-thalassemia minor does not influence the pregnancy outcome in the negative way significantly**.**
